# Organizational Commitment Among Residential Senior Care Workers

**DOI:** 10.1093/geroni/igaa057.068

**Published:** 2020-12-16

**Authors:** Tina Kilaberia

**Affiliations:** UC Davis Health, Sacramento, California, United States

## Abstract

Despite growing evidence of the increase in the aging population nationally, there continues to be a shortage of health and social care professionals who work with older adults. Some studies examine this phenomenon by looking at motivations that underlie commitment to geriatric careers. Others study commitment among those who are already geriatric professionals. Both the volume and diversity of the aging population challenge organizations to provide care. Drawing on 44 interviews, observations of 62 meetings, and a 5-year immersion, this organizational ethnography looks at commitment factors at a large, urban, faith-based residential senior care organization. Commitment factors are examined on three levels: daily tensions and rewards; value tensions and rewards; and deal breakers and clinchers. Findings show that intrinsic identity-based factors such as affective bonds with older persons and sharing in faith values sustain commitment on the person level. Interprofessional tensions may detract from commitment. Implications pertain to the role of leadership in equity-related and ethical tensions as well as the improved uptake of allied health professional expertises such as social work and chaplaincy. This study extends the extant knowledge by incorporating perspectives of social workers, chaplains, rehabilitation, recreational, diet and environmental services workers in addition to the more commonly examined groups such as nurses and certified nursing assistants, and in a setting that includes Assisted Living in addition to long-term care.

